# The development and psychometric properties of a new scale to measure mental illness related stigma by health care providers: The opening minds scale for Health Care Providers (OMS-HC)

**DOI:** 10.1186/1471-244X-12-62

**Published:** 2012-06-13

**Authors:** Aliya Kassam, Andriyka Papish, Geeta Modgill, Scott Patten

**Affiliations:** 1 Faculty of Medicine, Department of Community Health Sciences, Faculty of Medicine, University of Calgary, 3330 Hospital Drive NW, Calgary, AB, T2N 4 N1, Canada; 2 Department of Psychiatry, Department of Psychiatry, Faculty of Medicine, University of Calgary, 1403 - 29 Street NW, Calgary, Alberta, T2N 2T9, Canada; 3 Opening Minds Anti-Stigma Initiative, Mental Health Commission of Canada, Suite 800, 10301 Southport Lane SW, Calgary, Alberta, T2W 1S7, Canada; 4 Department of Community Health Sciences, University of Calgary, 3rd Floor TRW, 3280 Hospital Drive NW, Calgary, T2N 4Z6, Canada

## Abstract

**Background:**

Research on the attitudes of health care providers towards people with mental illness has repeatedly shown that they may be stigmatizing. Many scales used to measure attitudes towards people with mental illness that exist today are not adequate because they do not have items that relate specifically to the role of the health care provider.

**Methods:**

We developed and tested a new scale called the Opening Minds Scale for Health Care Providers (OMS-HC). After item-pool generation, stakeholder consultations and content validation, focus groups were held with 64 health care providers/trainees and six people with lived experience of mental illness to develop the scale. The OMS-HC was then tested with 787 health care providers/trainees across Canada to determine its psychometric properties.

**Results:**

The initial testing OMS-HC scale showed good internal consistency, Cronbach’s alpha = 0.82 and satisfactory test-retest reliability, intraclass correlation = 0.66 (95% CI 0.54 to 0.75). The OMC-HC was only weakly correlated with social desirability, indicating that the social desirability bias was not likely to be a major determinant of OMS-HC scores. A factor analysis favoured a two-factor structure which accounted for 45% of the variance using 12 of the 20 items tested.

**Conclusions:**

The OMS–HC provides a good starting point for further validation as well as a tool that could be used in the evaluation of programs aimed at reducing mental illness related stigma by health care providers. The OMS-HC incorporates various dimensions of stigma with a modest number of items that can be used with busy health care providers.

## Background

Mental illness related stigma can lead to low rates of seeking help, lack of access to care, under-treatment [1], and social marginalization [2]. Ultimately this leads to the inability of a person with mental illness to recover. Recovery is a process which occurs when people with mental illness discover, or rediscover, their strengths and abilities for pursuing personal goals and develop a sense of identity that allows them to grow beyond their mental illness [3,4].

Studies have varied in the dimensions of stigma examined, but the most common ones are: ‘perceived stigma’, which refers to one’s belief that others perceive an individual as socially unacceptable [4-7] and ‘self-stigma’, which refers to a similar, internalized perception of oneself leading to the fear of seeking help or disclosing one’s mental illness due to the stigma associated with mental illness [8]. Other dimensions of stigma that have been examined include social distance, which refers to one’s desire to maintain distance from people with mental illness [5,9], ‘dangerousness”, which refers to one’s belief that the individual is dangerous [6], recovery, which refers to one’s belief that people with mental illness can recover [3]. Emotional reactions [10] such as a lack of social responsibility as well as a lack of empathy or compassion towards people with mental illness are also dimensions of stigma [10]. While some may consider that compassion and social responsibility may be paternalistic and stigmatizing towards people with mental illness [11], these dimensions can be seen as important indicators of non-stigmatizing attitudes and are acknowledged widely as significant competencies of health care providers [4,12].

Evidence for an association between various dimensions of stigma is either lacking or largely inconsistent. For example, perceived stigma has been found to be unrelated to help seeking in some studies [13,14]. It must also be noted that most of the models that currently describe the dimensions related to mental illness stigma are based on individual-level rather than a sociological-level of structural indicators of stigma which can occur at the institutional or government level. For the purposes of this paper, we will focus on individual-level of stigma, specifically by health care providers.

Stigma has been criticized as being too vaguely defined and individually focused [15] and thus existing models have defined stigma as a dynamic interrelationship of components. This interrelationship involves cognitive, affective and behavioural components. In order to understand and measure stigma it is important to conceptualize the term within a more detailed model.

A model by Link and Phelan which has been widely used in the literature describes mental illness stigma as four components which are: labelling, stereotyping, separating and status loss/discrimination. Throughout these components, it has been suggested that emotional reactions can occur [15]. There are three cognitive processes; labelling begins when personal characteristics are signalled to show an important difference between the person who stigmatises and the stigmatised. Stereotyping then occurs when the labelled differences are associated with undesirable characteristics. This is followed by categorically distinguishing or separating between the mainstream group and the labelled group, perceiving the labelled group as fundamentally different. Ultimately, the labelled group is then devalued, rejected and excluded through the process of status loss or discrimination [15].

Another model by Corrigan, contains the components of stereotype, prejudice and discrimination that are seen as causally related. For example, a person who believes (cognition) a person with mental illness is dangerous (stereotype) might negatively evaluate or fear (affect) the person with mental illness as dangerous, leading to prejudice. This would then lead to discrimination (behaviour) when the person is treated inappropriately for their mental illness by receiving sub-standard care [16].

What can be drawn from these models so far is that they have commonly embedded within them the cognitive, affective and behavioural components. While they are useful in describing mental illness related stigma as a social psychological phenomenon, they may not be valid or useful for measuring outcomes in mental health promotion and health education interventions aimed to reduce the stigma of mental illness.

A final conceptualization of stigma is the tri-partite model, which proposes that stigma is an overarching term including three core elements: knowledge (misinformation/differences in understanding due to culture or religion), attitudes (prejudice) and behaviour (discrimination) [17]. The knowledge, attitudes and behaviour framework allows clear intervention targets and units of measurement [17,18]. The importance of knowledge, attitudes and behaviour has been established in medical education with medical students, nurses, and other health care providers [17-20]. The knowledge, attitudes and behaviour framework is also one that is widely used in health promotion [21,22]. Similar to the Corrigan model, the tri-partite model focuses on the problem of attitudes in the form of prejudice which can be elicited as common stereotypes or emotional reactions rather than separating them like the previous models. The tri-partite model is adaptable in that it allows for attitudes towards people with mental illness to be comprised of the various dimensions of stigma [17].

In the literature, it has been shown that attitudes towards people with mental illness can be measured using stereotypes such as: ‘people with mental illness are dangerous,’ and ‘people with mental illness do not recover’ [23,24] as well as a desire for social distance because of the aforementioned stereotypes [7]. Stigmatizing attitudes can also be measured in the form of emotional reactions towards people with mental illness. Finally, disclosing that one has a mental illness, because of the dimensions described above, can lead to self stigma and may also be an indicator of mental illness related stigma [24,25]. While it has been shown that self stigma is different than holding stigmatizing attitudes towards people with mental illness [25], we believed it was important to measure in health care providers because, we saw disclosure as a dimension of stigma that would also indicate whether the respondent held stigmatizing attitudes towards mental illness. For example, those who would disclose that they had a mental illness may not think that mental illness is something to be ashamed of and may therefore be less stigmatizing. This has been described in the literature where some refuse to be diminished by stigma and becoming more active participants of change in health care [7]. Also, potential users of the instrument, such as professional organizations, are likely to be interested in the issue of disclosure because of a desire to see their members receive appropriate treatment and support for mental health issues.

Through extensive review of the academic literature on surveys used to measure attitudes towards people with mental illness, a large gap was shown in the area of surveys used to measure the attitudes of health care providers [26]. A new measure of stigma intended for healthcare providers is pertinent because stigma among health care providers differs from other kinds of stigmas held by various other groups. For example, it has been reported that people with mental illness have poorer physical health in part because medical professionals wrongly associate the physical symptoms experienced by the person with mental illness to the mental illness itself [27-30]. This could be due to a phenomenon called ‘diagnostic overshadowing’ [17,30].

Diagnostic overshadowing can be defined as the process by which the physical problems of a patient are over-shadowed by their psychiatric diagnosis [17,30]. It is important to note that diagnostic overshadowing is not unique to primary care and may occur in other areas of health services [17,26]. An investigation of the physical healthcare of patients with schizophrenia in primary care [31] showed that people with schizophrenia were no more likely than the general population to be targeted for physical health checks despite increased physical health risks. Furthermore, people with schizophrenia were significantly less likely to receive important basic health checks such as blood pressure and cholesterol measurement [31].

Medical practitioners also diagnose and treat people with mental illness differently. For example, people with mental illness are “substantially less likely to undergo coronary re-vascularisation procedures” compared to people without mental illness [32]. Similarly, people with co-morbid mental illness and diabetes are less likely to be admitted to hospital for diabetic complications than those with no mental illness [33].

Furthermore, people with mental illness may have less access to medical care [34,35] such as obtaining a primary care physician [34,35] as there is a need for community mental health centres to address barriers to primary medical care [36]. People with mental illness may also feel unwelcome in certain medical settings because of staff attitudes [36-42].

Although health care providers are thought to hold attitudes that are positive, compassionate and encouraging towards people with mental illness, this is often not the case. Health care providers may be ignorant about the possible outcomes of people with mental illness. Often this may be due to inadequate training [36]. It has been reported that 68% of the mental health professionals surveyed thought that most clinicians do not receive appropriate training in dealing with people with severe mental illness [37].

A large body of research on the attitudes of health care providers has repeatedly shown negative attitudes towards people with mental illness. This has been a problem and continues to be a problem in primary care, mental health services and within the education of health care providers [37-42].

As part of its 10-year mandate, The Mental Health Commission of Canada (MHCC) has embarked on an anti-stigma initiative called Opening Minds (OM) to change the attitudes and behaviours of Canadian health care providers towards people with a mental illness. OM is the largest systematic effort undertaken in Canadian history to reduce the stigma and discrimination associated with mental illness. OM’s philosophy is to build on the strengths of existing programs from across the county, and to scientifically evaluate their effectiveness. A key component of programs being evaluated is contact-based educational sessions, where target audiences hear personal stories from and interact with individuals who have experience with mental illness and have recovered or are managing their illness. OM’s goal is to replicate effective programs nationally, develop new interventions to address gaps in existing programs and add other target groups over time. (http://www.mentalhealthcommission.ca/English/Pages/OpeningMinds.aspx)

Evaluation describes and explains the practice of participants to determine their effectiveness [43,44]. Because evidence-based educational interventions are given priority, evaluation drives undergraduate, post graduate and continuing education curriculum development [44]. In order to achieve its goal with healthcare providers, the Opening Minds initiative required a current, reliable and valid tool to evaluate best practices to reduce the stigma of people with mental illness. Many of the evaluative tools that exist today are not adequate for our purpose because they do not relate specifically to the role of the health care provider.

The aim of this study was to develop, and test the Opening Minds Scale for Healthcare Providers (OMS-HC).

## Methods

Research carried out in this study is in compliance with the Helsinki Declaration (http://www.wma.net/e/policy/b3.htm). This study was approved by the Conjoint Health Research Ethics Board of the University of Calgary, Ethics ID E22724.

### Development phase

The development of the OMS-HC began by generating an item pool of items pertaining to the dimensions of stigma described earlier from existing scales as well as new items that were developed by the researcher (AK) from consultations with people working with programs to reduce the stigma of mental illness by health care providers. What resulted was a pool of 50 items with five dimensions to measure stigma which were 1.) Recovery, 2.) Social responsibility, 3.) Social distance 4.) Other concepts (dangerousness, diagnostic overshadowing) 5.) Disclosure. The initial items and the scales from which they were derived are provided (see Additional file 1). These questions were then sent to three people who had a mental illness for their feedback. The questions were also reviewed by a research team which consisted of a sociologist, psychiatrist, anti-stigma program manager, and research associate. From this collective feedback, survey questions were eliminated and amended resulting in a 42 question scale.

Cognitive interviewing was conducted with the 42 questions and based on this feedback the scale was reduced to 26 questions. The individual interviews were 20 minutes in length. Cognitive interviewing is a method of evaluating sources of response error in survey questionnaires that focuses mainly on the questionnaire and the cognitive processes that respondents use to answer the survey [45]. For the OMS-HC scale, cognitive interviews were conducted by one of the authors (AK). For the conduct of the cognitive interviews, six volunteers were recruited using a convenience sampling method. Volunteers all worked in the health care field and were female. Questions were asked around what the volunteer felt each item on the questionnaire was referring to, whether they felt pressured to respond to the item in a certain manner (positively or negatively) and whether the item made sense and was measuring attitudes of health care providers towards people with mental illness [45].

The questions were then reviewed again by the research team and one question was eliminated because it was not judged to measure stigma, leading to an initial version of the OMS-HC. The initial OMS-HC scale had 25 questions as well as additional demographic questions. The scale used definitions of mental health problems/illnesses, recovery and health care system drawn from the MHCC framework document [46].

Seven focus groups of health care providers were then held to ask about the wording of the scale, suggest new items as well as to explore their attitudes towards people with mental illness. A focus group of people with mental illness was conducted to ensure the language and tone of the scale was appropriate and that no concepts were missing. For this part of the study, we invited volunteer participants who were health care providers or health care providers in training in Calgary to participate in the focus groups. Focus groups consisted of 6–10 people. For the focus groups, we aimed to recruit participants for each of the following disciplines:

· General practitioners

· Other physicians including psychiatrists

· Surgeons

· Nurses

· Psychiatric nurses

· Medical students

· Social workers/social worker students

· Occupational therapists/occupational therapy students

· Psychologists

· Pharmacists

Focus groups were selected using a convenience sampling method. Emails were sent to local mental health community organizations as well as hospitals and clinics. Medical students and doctors were recruited through the author’s (AP) contacts. Several concepts that were explored in the focus groups were the use of the term “mental illness” as opposed to a specific diagnosis of mental illness such as depression, the definition of mental illness, the definition of recovery, the social desirability bias and the relevance of the items to health care providers. The focus groups took place one after another and the scale presented to each focus group was amended based on the previous focus group’s feedback so that the scale would be improved each time. Focus group data was then transcribed and analyzed for each focus group transcript. The researcher (AK) looked for consensus in qualitative responses around the term “mental illness” the definition of mental illness, the definition of recovery, social desirability bias and the relevance of the items to health care providers [47]. If the majority (greater than 50% of the focus group members agreed on a concept, then the researcher made changes to the final scale accordingly. These changes resulted in a reduction from 25 to 20 items.

### Testing phase

In this part of the study, we identified participants by searching for health regions, professional organizations and academic departments affiliated with health care providers or health care providers in training to participate in completing the survey.

Participants were contacted locally, provincially and nationally by e-mail for the surveys which were conducted online. Faculty deans and department heads at the Universities for the areas of medicine, nursing, social work, occupational therapy and psychology were contacted in order to access students in each group. Professional associations were also contacted across Canada to request members who are health care providers to complete the questionnaire. The questionnaire was available online for respondents to complete. Any health care provider or trainee was eligible to participate in the study if they provided informed consent to participate.

We also sought to determine the relationship between the scale items and social desirability items. The social desirability bias can be defined as the tendency of individuals to report favourable impressions of themselves on measures that ascertain sociological and psychological variables of interest such as attitudes to mental illness, ethnic minorities and gender. If items of the scale correlate highly with scores of social desirability, the scale could be influenced by social desirability bias [47].

All statistical analysis was carried out using STATA version 12.0. The Spearman's rank correlation coefficient or Spearman's rho (ρ) was used to determine the relationship between total scores and the Marlow-Crowne Social Desirability scale and the total scores of 669 health care providers. This particular correlation coefficient is considered to be more robust and does not make restrictive assumptions about the frequency distribution of the variables [48].

Test-retest reliability of the 20-item scale was conducted with a sub-sample of health care providers who consented to be contacted four to six weeks after the first time they completed the survey. The intraclass correlation comparing the total scores at the two time periods was determined.

An item analysis was conducted and the Cronbach’s alpha was computed to determine the overall consistency of the scale. Item-total correlations were calculated along with a factor analysis of the items. Those items that did not correlate strongly with the total score were eliminated. Factor analysis was conducted using principal component analysis. The methods used for retaining factors were keeping those factors that had Eigen values above 1.0, and had more than four items that loaded onto a single factor.

## Results

**Figure 1:**
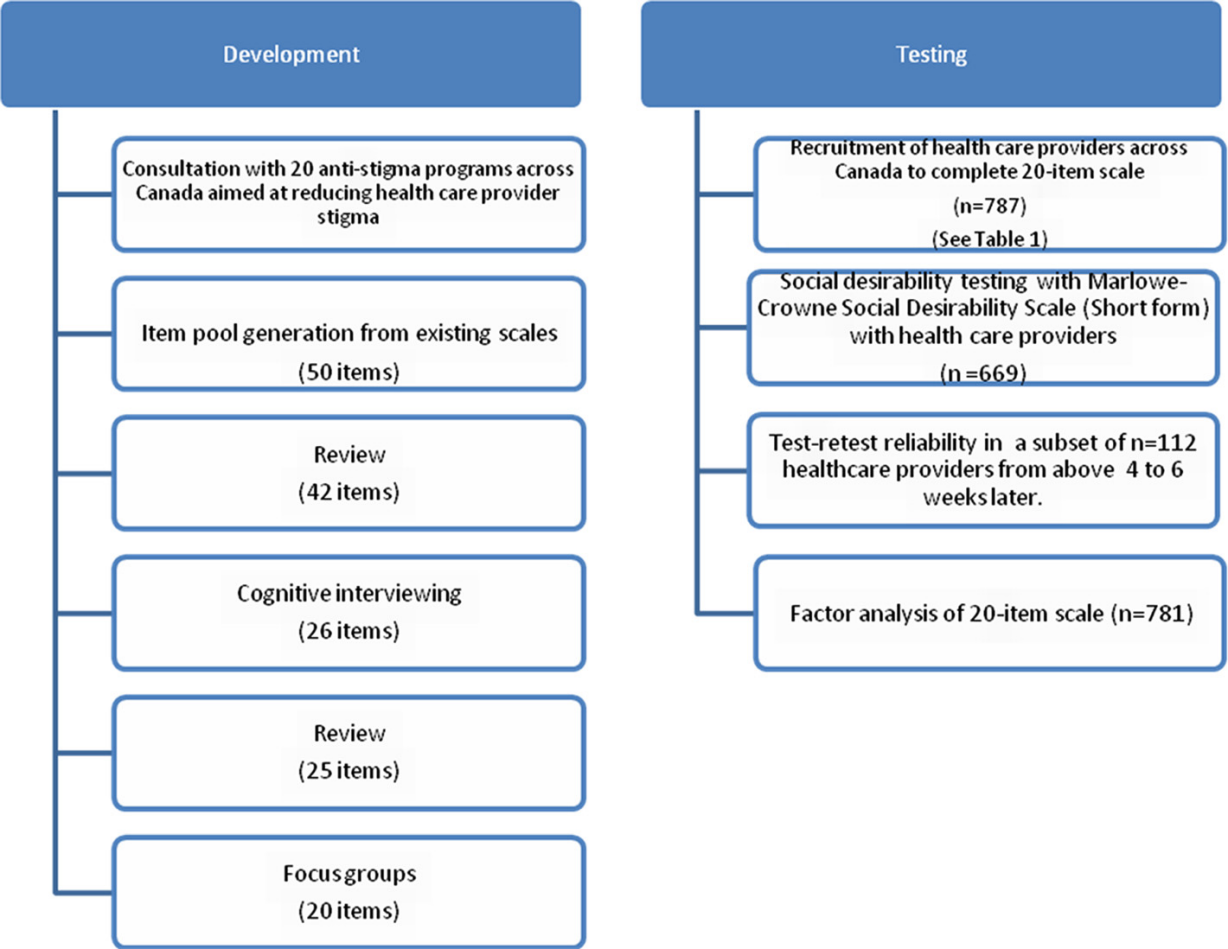
The development and testing of the OMS-HC.

Figure 1 shows the development and testing of the scale. The review process with the research team, cognitive interviewing and focus groups during the development phase led to a honed-in scale for the testing phase. The number of items was modified after each of the processes during development and led to a refined scale (the number of items is shown in the brackets, see Figure 1). Several of the items were new and developed by the research team. Through the development process, items were re-worded or removed and new items were also added from another scale called the Medical Condition Regard Scale [49] which was recommended by a research team member after the initial item pool was reviewed. The Medical Condition Regard Scale is a non-condition-specific scale developed from the literature on physicians' responses to patients they like and dislike, stigma definitions, and discussions with primary care faculty.

### Development

Seven health care provider focus groups with 64 health care providers/trainees were held and consisted of 16 males and 48 females. Focus group participants were 22 to 65 years of age. In total, there were 14 medical students, 2 psychiatrists, 8 psychiatry residents, 4 physiotherapists, 9 psychologists, 6 recreational therapists, 10 social workers, 2 nurses and 9 occupational therapists. In the focus group of people with lived experience of mental illness, there were 5 males and 1 female and all were currently diagnosed with a mental illness.

Major discussion topics in the focus groups circled around whether or not to use the term “mental illness”, whether or not to include a definition of the term “mental illness”, the wording of each of the items, whether or not to include a specific diagnosis or to specify the severity of mental illness. Overall, there was no consensus on any of these topics and ultimately the main value of the focus groups was helpful feedback on individual items in terms of wording and retention. A suggestion for an item that came from the focus group of people with mental illness was to determine whether health care providers felt that medication was the best treatment of mental illness. This is because members of this focus group reported feeling stigmatized by health care providers because they were viewed as being more likely to over-medicate patients and less likely to listen or provide other forms of therapy. The use of medication was also perceived as a means of increasing social distance.

From a 25-item scale, a 20-item scale resulted after the focus groups. Regarding severity, the term “managed” was added to one item to imply appropriate treatment was being received, even if the illness was severe. The term “managed” was included so as not to evoke responses which would lead to ceiling or floor effects. The testing version of the scale and the item sources are also provided (see Additional file 2). It must be noted that the items shown may differ in how they are worded from the original item because these were discussed in focus groups and changed according to suggestions made in the focus groups.

#### Testing

Seven hundred and eighty seven health care providers responded to the online survey of which n = 781 provided complete data. Table 1 shows the demographic characteristics of the group (n = 787) as well as total and subscale scores to be discussed later. In the overall group, most respondents were aged between 26 and 64 years, were white, female, had personal contact with a person who had a mental illness, had treated a person with mental illness and resided in British Columbia, Alberta, Manitoba or Ontario. The majority of respondents were medical students, psychiatric nurses, registered nurses, social workers or other types of health care providers such as pharmacists, recreational therapists, counselors etc.

**Table 1:** **Demographic characteristics of healthcare providers with total and subscale scores**^**b**^

**Demographic variables**	**Health care providers including medical students, Frequency (%) n = 787**	**Total score**	**Attitudes subscale**	**Disclosure subscale**
		**(95% C.I.)**	**(95% C.I.)**	**(95% C.I.)**
**Age (years)**				
18–25 years	106 (13.5)	60.7	14.9	16.3
		(59.6–61.8)	(14.1–15.7)	(15.8–16.8)
26–44 years	349 (44.4)	57.7	12.8	15.1
		(57.2–58.2)	(11.8–12.5)	(14.9–15.4)
45–64 years	319 (40.5)	56.3	11.3	14.4
		(55.8–56.8)	(10.9–11.6)	(14.2–14.7)
65 years or older	13 (1.6)	55.5	10.1	13.8
		(53.4–57.7)	(8.7–11.5)	(12.3–15.2)
**Sex**				
Female	631 (80.2)	57.3	11.9	14.9
		(56.9–57.6)	(11.6–12.1)	(14.7–15.1)
Male	156 (19.8)	58.5	13.2	15.3
		(57.6–59.5)	(12.5–13.9)	(14.8–15.7)
**Do you know a close friend or family member with mental illness?**				
Yes	634 (80.6)	57.3	11.9	14.9
		(56.9–57.7)	(11.6–12.1)	(14.7–15.1)
No	130 (16.5)	58.4	13.2	15.5
		(57.6–59.1)	(12.6–13.8)	(15.1–15.9)
Don’t know	23 (2.9)	58.9	13.9	15.5
		(57.3–60.4)	(12.5–15.2)	(14.5–16.6)
**Have you ever treated a person with a mental illness?**				
Yes	657 (83.5)	57.2	11.7	14.9
		(56.8–57.5)	(11.5–12.0)	(14.7–15.0)
No	113 (14.3)	59	14	15.7
		(58.1–59.9)	(13.3–14.7)	(15.3–16.1)
Don’t know	17 (2.2)	60.8	15	15.4
		(56.8–64.7)	(11.9–18.1)	(13.9–16.8)
**Ethnicity**				
White	683 (86.8)	57.3	11.8	14.9
		(56.9–57.6)	(11.6–12.1)	(14.7–15.1)
Asian	43 (5.5)	59.4	15	15.8
		(58.1–60.8)	(14.0–16.1)	(15.0–16.6)
South East Asian	32 (4.1)	58.5	13.8	15.7
		(56.8–60.2)	(12.6–15.0)	(15.0–16.4)
Aboriginal	12 (1.5)	58.5	14.3	14.3
		(52.1–64.9)	(9.6–18.9)	(12.4–16.3)
Black	8 (1.0)	56.6	11.8	14.5
		(52.9–60.3)	(9.9–13.6)	(12.1–16.9)
Arab/West Asian	7 (0.9)	62.7	15	17.1
		(60.0–65.5)	(11.8–18.2)	(14.1–20.2)
Latin American	2 (0.2)	58	11	17
		(45.3–70.7)	(-27.1–49.1)	(-8.4–42.4)
**Province**				
Ontario	204 (25.9)	56.6	11.2	14.8
		(56.1–57.2)	(10.8–11.6)	(14.4–15.1)
Manitoba	203 (25.8)	56.8	11.7	14.4
		(56.3–57.4)	(11.2–12.1)	(14.1–14.7)
Alberta	195 (24.8)	59.5	13.8	15.8
		(58.8–60.2)	(13.3–14.3)	(15.5–16.2)
British Columbia	153 (19.4)	57	11.9	15
		(56.2–57.8)	(11.3–12.5)	(14.6–15.4)
Atlantic provinces	21 (2.7)	57	11.2	14.2
		(55.3–58.6)	(9.9–12.4)	(13.1–15.4)
Northwest Territories and Yukon	6 (0.8)	62	14.8	16.7
		(44.3–79.7)	(4.1–25.6)	(13.1–20.2)
Quebec	4 (0.5)	58	12.8	15.3
		(56.2–59.8)	(7.2–18.3)	(10.0–20.5)
Saskatchewan	1 (0.1)	-	-	-
**Professional group**				
Social workers/social work students	167 (21.2)	56.6	11	14.8
		(56.0–57.3)	(10.6–11.5)	(14.4–15.2)
Nurses/nursing students	138 (17.5)	57.3	12.3	14.8
		(56.5–58.1)	(11.7–12.9)	(14.4–15.2)
Physicians and medical students	129 (16.4)	61	15.1	16.4
		(60.2–61.8)	(14.5–15.7)	(16.0–16.8)
Psychiatric nurses/psychiatric nursing students	108 (13.7)	55.7	11	14.4
		(55.0–56.5)	(10.4–11.5)	(13.9–14.8)
Other	102 (13.0)	57.2	11.8	14.5
		(56.2–58.1)	(11.1–12.6)	(14.0–15.0)
Psychologists/graduate students in psychology	95 (12.1)	57.1	11.8	14.9
		(56.0–58.2)	(11.1–12.5)	(14.4–15.4)
Occupational therapists/ occupational therapy students	48 (6.1)	57.4	11.4	14.9
		(56.3–58.5)	(10.6–12.3)	(14.2–15.5)

^b^Higher scores indicate more stigma.

### Scoring the scale

The testing version of the OMS-HC contained 20 items. A 5-point Likert scale was used and response options were 1 = Strongly disagree, 2 = Disagree, 3 = Neither agree nor disagree, 4 = Agree and 5 = Strongly agree. Scores could range from 20 to 100 and a lower score indicated less stigma. Items 3, 8, 9, 10, 11, 15, 19 required reverse scoring. The 12 item scale had scores ranging from 12 to 60, and only one of the 12-items was reverse scored. Regarding the subscales, (see results on factor analysis), the scoring of the attitudes of healthcare providers towards people with mental illness subscale may range from 7 (least stigmatizing) to 35 (most stigmatizing) while the scoring for the attitudes towards disclosure of a mental illness subscale may range from 5 (least stigmatizing) to 25 (most stigmatizing).

The mean total score for the 20 item scale for all health care providers (see Table 1) was 57.5 (95% C.I. 57.2 – 57.9). Scores ranged from 41.0 to 96.0 and the standard deviation was 4.8.

The mean total score for the 12 item scale was 27.0 (95% C.I. 26.7 - 27.4), scores ranged from 16.0 to 67.0 and the standard deviation was 5.0. The mean total score for the attitudes of healthcare providers towards people with mental illness subscale was 12.1 (95% C.I. 11.9 to 12.4) and for disclosure was 15.0 (95% C.I. 14.8 to 15.2). Scores ranged from 7.0 to 35.0 (SD = 3.6) for the attitudes of healthcare providers towards people with mental illness and from 8.0 to 23.0 (SD = 2.5) for attitudes towards disclosure of a mental illness.

Within each demographic group, there did not appear to be differences in scores except that among the age groups, the 18–25 year age group score was slightly higher than that of the other groups as was the score of health care providers from Alberta and the physicians/medical student group. This may be explained by the fact that the physician/medical student group consisted largely of medical students aged between 18–25 who were from Alberta. These 2^nd^ year medical students (n = 112) were recruited as part of another study which involved the evaluation of an educational intervention aimed at reducing stigma and will be described elsewhere. The medical students completed the 20-item OMS-HC as a baseline assessment prior to a psychiatry course. The Cronbach’s alpha reliabilities for the medical student sample remained internally consistent (0.78 for the 20-item scale and 0.71 for the 12 item scale) and the subscales were responsive to change.

### Item total correlations

An optimal item-total correlation was considered to be between 0.2 and 0.5 [50]. Six of the 20 items had item-total correlations lower than 0.2 (see Table 2).

**Table 2:** Item-total correlations of each scale item and total score (20 items on OMS-HC)

**1. I am more comfortable helping a person who has a physical illness than I am helping a person who has a mental illness.**	.514^**^
2. If a person with a mental illness complains of physical symptoms (e.g. nausea, back pain or headache), I would likely attribute this to their mental illness.	.422^**^
3. If a colleague with whom I work told me they had a managed mental illness, I would be as willing to work with him/her.	-.072^*^
4. If I were under treatment for a mental illness I would not disclose this to any of my colleagues.	.426^**^
5. I would be more inclined to seek help for a mental illness if my treating healthcare provider was not associated with my workplace.	.350^**^
6. I would see myself as weak if I had a mental illness and could not fix it myself.	.572^**^
7. I would be reluctant to seek help if I had a mental illness.	.516^**^
8. Employers should hire a person with a managed mental illness if he/she is the best person for the job.	**-.016**
9. I would still go to a physician if I knew that the physician had been treated for a mental illness.	-.078^*^
10. If I had a mental illness, I would tell my friends.	-.205^**^
11. It is the responsibility of health care providers to inspire hope in people with mental illness.	**.011**
12. Despite my professional beliefs, I have negative reactions towards people who have mental illness.	.475^**^
13. There is little I can do to help people with mental illness.	.466^**^
14. More than half of people with mental illness don’t try hard enough to get better.	.407^**^
15. People with mental illness seldom pose a risk to the public.	-.072^*^
16. The best treatment for mental illness is medication.	.232^**^
17. I would not want a person with a mental illness, even if it were appropriately managed, to work with children.	.317^**^
18. Healthcare providers do not need to be advocates for people with mental illness.	.289^**^
19. I would not mind if a person with a mental illness lived next door to me.	-.129^**^
20. I struggle to feel compassion for a person with a mental illness.	.400^**^

### Test-retest reliability

A subset of n = 112 participants from the original sample of participants consented to complete the survey again. This subset of n =112 is different from the n =112 medical students discussed later. The intra-class correlation coefficient was 0.66 (95% CI 0.54 to 0.75, p < 0.001) for the total scores indicating near satisfactory test-retest stability as an intra-class coefficient of 0.7 or higher is considered satisfactory [50,51].

The demographic characteristics of the sample (n = 112) that responded to the OMS-HC for the test-retest reliability were similar to the entire sample. Again, the majority were white, female, nurses or social workers aged 26–64 years from British Columbia, Alberta, Manitoba and Ontario.

### Social desirability bias testing

The total score on the OMS-HC was correlated with the total score of the Marlowe-Crowne Social Desirability scale (short form) for a sample of health care providers (n = 669). There was a small but significant correlation, ρ = 0.10, (95% CI 0.08 to 0.13, p = 0.01).

### Factor analysis

From the item analysis, six items were shown to have poor item-total correlations below 0.2. After removal of these items, a factor analysis revealed a three factor structure accounting for 48% of the total variance explained, with Eigen values 4.0, 1.7 and 1.0 respectively. Given the Eigen value of exactly 1.0 (as opposed to being greater than 1.0) for the third factor and that only two items loaded onto it, (one of which also loaded to the first factor) we decided to eliminate these items (16 and 17) and re-run the factor analysis. What resulted was a two-factor solution of 12 items accounting for 45% of the variance (see Table 3). Factor 1 explained 24.8% of the total variance and factor 2 explained 23.1% of the total variance. Although the analysis did not reveal a single factor structure, the two factor structure yielded two aspects of mental illness related stigma being assessed within the scale. These appeared to refer to: 1) attitudes of healthcare providers towards people with mental illness (7 items) 2) attitudes of healthcare providers towards disclosure of a mental illness (5 items). All items had factor loadings > 0.5, with all items clearly loading onto either of the two factors (see Table 3).

**Table 3:** Factor analysis of remaining 12 items of the OMS-HC

**Cronbach’s alpha = 0.78**	**Factor Loading**
**Attitudes of health care providers towards people with mental illness, Cronbach’s alpha = 0.75**	
· I am more comfortable helping a person who has a physical illness than I am helping a person who has a mental illness. (Item 1 of original scale)	0.61
· If a person with a mental illness complains of physical symptoms (e.g. nausea, back pain or headache), I would likely attribute this to their mental illness. (Item 2 of original scale)	0.51
· Despite my professional beliefs, I have negative reactions towards people who have mental illness. (Item 12 of original scale)	0.67
· There is little I can do to help people with mental illness. (Item 13 of original scale)	0.75
· More than half of people with mental illness don’t try hard enough to get better. (Item 14 of original scale)	0.62
· Healthcare providers do not need to be advocates for people with mental illness. (Item 18 of original scale)	0.56
· I struggle to feel compassion for a person with a mental illness. (Item 20 of original scale)	0.64
**Attitudes of health care providers towards disclosure of a mental illness, Cronbach’s alpha = 0.72**	
· If I were under treatment for a mental illness I would not disclose this to any of my colleagues. (Item 4 of original scale)	0.76
· I would be more inclined to seek help for a mental illness if my treating healthcare provider was not associated with my workplace. (Item 5 of original scale)	0.61
· I would see myself as weak if I had a mental illness and could not fix it myself. (Item 6 of original scale)	0.60
· I would be reluctant to seek help if I had a mental illness. (Item 7 of original scale)	0.67
· If I had a mental illness, I would tell my friends. (Item 10 of original scale)	0.70

### Internal consistency

The Cronbach’s alpha of the 20-item scale was 0.82. After removal of the items during factor analysis cronbach’s alpha was 0.78 (0.75 and 0.72 for the attitudes towards people with mental illness subscale and attitudes towards disclosure of a mental illness subscale respectively). An alpha greater than 0.70 was considered acceptable [52]. When correlating the subscales, the attitudes towards mental illness subscale was moderately correlated with the subscale pertaining to attitudes towards disclosure, ρ = 0.30, (95% C.I. 0.23 to 0.36, p <0.001).

## Discussion

Until this study, a scale had not been developed in Canada to assess the attitudes of health care providers towards people with mental illness. We have developed and tested a scale that we subsequently found to have good internal consistency and adequate test-retest reliability during its testing phase. The development of the scale was guided by the tri-partite model of stigma arising from literature reviews focusing on the stigma and how attitudes towards people with mental illness can be measured. We chose to adopt the tri-partite model because this allows for the measurement of clear outcomes such as attitudes which can be measured using the various dimensions of stigma.

Extensive stakeholder consultations were held with regards to the items to be included in the scale and items included the dimensions of recovery, social responsibility and social distance. Other dimensions of stigma such as dangerousness, treatment of people with mental illness and disclosure of a mental illness were also seen as important and items pertaining to them were included in the scale.

The scale did not correlate highly with a short form of the Social Desirability scale. This indicates that while there may be some relationship between measuring stigma and social desirability (i.e. people responding to the instrument may attempt to provide socially desirable responses), the scale responses were not strongly related to social desirability. Social desirability can be a threat to validity of scales such as this one.

As with any scale, consensus on what to measure and the wording of items is difficult to obtain especially in the area of mental illness where there are different schools of thought in treatment and recovery. Through our study, which used thorough methods from developing to testing the OMS-HC, we believe that we obtained views from several types of health care providers from the focus groups and tested the scale across Canada to obtain a wide range of professions, age groups as well as representation of sex and province. Although we did not achieve fully comprehensive representation of professions such as medical doctors, provinces such as the northern, central, French speaking, and maritime parts of Canada, our sample was in many respects broadly representative of diverse health care provider groups. This was the first attempt to develop a tool to assess attitudes for health care providers in Canada.

The total scores were slightly higher in the 18–25 age group, those in the physician/medical student group and those in Alberta. This requires further exploration and future research can look into closely examining other variables that may influence scores on the OMS-HC such as socio-demographic variables. The factor analysis showed two subscales which measured attitudes towards people with mental illness using the various dimensions of stigma and attitudes towards disclosure and help seeking. While the 12-item scale accounts for only 45% of the variance, this scale is succinct in its number of items which would offset response burden and increase the feasibility of its use in busy health care providers. Future research could also examine testing more items along with those tested in this study to determine if these change the variance accounted for by the two factors or if other factors emerge with sufficient items for additional subscales. Factor analysis in each health care provider group may also provide insight into subscales which may be unique to certain health care provider groups.

We adopted the idea that stigmatizing attitudes can also be measured in the form of disclosing whether one has a mental illness and/or seeking help for it, because self stigma may also be an indicator of mental illness related stigma [24,25]. Likewise, those who would disclose that they had a mental illness may not think that mental illness is something to be ashamed of and may therefore be less stigmatizing to other people with mental illness. In support of the literature that supports self stigma as distinct from holding stigmatizing attitudes towards people with mental illness [25], we found that while health care provider’s attitudes towards disclosure were related to their attitudes towards people of mental illness, there was only a moderate correlation between these two constructs. Evidence for this was also shown by our factor analysis which yielded a two factor solution for the OMS-HC in which we had a 7 item subscale measuring attitudes towards people with mental illness and a 5 item subscale measuring attitudes towards disclosure of a mental illness.

As such, the overall scale should be used with caution given the relatively low between factor correlation. Nonetheless, there is a possibility that the OMS-HC may behave differently in different groups of health care providers so we advise using the overall scale as well as subscale scores to gain further insight into participant responses. Attitudes towards disclosure and help seeking are important to measure in health care providers as this provides an indication of the stigma that they believe exists due to having a mental illness and how this would impact seeking help. As health care providers are not immune to mental illness themselves, this is an important dimension of stigma to measure in relation to health care provider well being [53].

As there is no gold standard for assessing attitudes of Canadian health care providers towards people with mental illness, we hope that this scale provides a good starting point for further testing and development as well as a pilot scale that can be used in the evaluation of programs aimed at reducing the stigma of mental illness in health care providers. This scale can be used both in the realms of education such as evaluating courses in health care programs from undergraduate to postgraduate areas of health care providers, continuing professional development as well as part of quality improvement and quality assurance initiative within hospitals and health care regions.

Future research with the OMS-HC should also include its use in evaluating future mental illness anti-stigma related educational interventions, validating it in French so that it can be used in French speaking areas of Canada and validating it with physicians across Canada. Other areas of future research could incorporate additional instruments for external validity such as those that measure knowledge [54] intended behaviour [55] or behavior such as Objective Structured Clinical Exams (OSCEs) used in teaching health care providers to determine the relationship between the tri-partite model components of knowledge, attitudes and behavior. Further research in determining minimally important change (MIC) [56] (the meaning of changes in scores on the OMS-HC over time) is also warranted. This would provide valuable information to detect clinically important changes also known as the responsiveness of an instrument which is an aspect of validity [22].

## Conclusions

The OMS–HC provides a good basis for further validation as well as a tool that could be used in the evaluation of programs aimed at reducing mental illness related stigma by health care providers. Future research and testing is required for the OMS-HC pertaining to additional items and other variables that may influence responses of health care providers such as demographic variables. The OMS-HC incorporates various dimensions of stigma with a modest number of items that can be used with busy health care providers. While this project is supported by a national commission, it may have international applicability.

## Endnotes

^a^As consensus could not be reached, we decided to keep the term mental illness because we thought the term itself would evoke stigma. Where applicable in terms of severity, we added the word “managed” to the item pertaining to hiring a person with mental illness since they may be the best person for the job if their condition was managed. We felt that the term “mental illness” in itself would evoke a response regardless of severity.

## Competing interests

At the time this study was conducted AK was a post-doctoral fellow at the University of Calgary and her position was supported by the Mental Health Commission of Canada. As part of the fellowship, AK was a research associate with the Opening Minds Initiative for the Mental Health Commission of Canada. SP was the post-doctoral supervisor for AK. GM is currently a research associate with the Mental Health Commission of Canada. AP is a resident in Psychiatry at the Faculty of Medicine, University of Calgary.

## Authors’ contributions

AK designed and conducted the study. AP helped with data collection and designing the study. GM helped with data analysis. SP supervised the study. All authors were involved in writing and editing the manuscript. All authors read and approved the final manuscript.

## Pre-publication history

The pre-publication history for this paper can be accessed here:

http://www.biomedcentral.com/1471-244X/12/62/prepub

## Supplementary Material

Additional file 1 Initial Item Pool for the OMS-HC. Click here for file

Additional file 2 Testing version of the OMS-HC. Click here for file
