# Supplementary material for: The development and psychometric properties of a new scale to measure mental illness related stigma by health care providers: The opening minds scale for Health Care Providers (OMS-HC)
Source: BMC Psychiatry. 2012 Jun 13;12:62. doi: 10.1186/1471-244X-12-62 (PMC3681304; doi:10.1186/1471-244X-12-62)
Supplement: Additional file 1 — Initial Item Pool for the OMS-HC. [file 1471-244X-12-62-S1.docx]

Additional File 1: Initial Item Pool for the OMS-HC

Recovery

| 1. | Only people with mental health problems/illnesses who are considered well enough are suitable to participate in goal setting and monitoring for recovery (STARS) (R). |
| --- | --- |
| 2. | It is not realistic to expect most people with mental health problems/illnesses to recover (STARS) (R). |
| 3. | The goal setting and monitoring process of recovery is most effective when the health care provider makes all of the decisions (STARS) (R). |
| 4. | All people with mental health problems/illnesses can strive for recovery (RAQ-7). |
| 5. | Recovery cannot occur if symptoms of mental health problems/illnesses are present (RAQ-7) (R). |
| 6. | Recovering from the consequences of mental health problems/illnesses is sometimes more difficult than recovering from mental health problems/illnesses themselves (RAQ-16). |
| 7. | One role for the health care provider is to foster hope for recovery (new). |
| 8. | Family and friends do not play as an important role as the health care provider in the recovery process of people with mental health problems/illnesses (new) (R). |
| 9. | It is important for people in recovery to choose from a variety of treatment options (i.e., individual, group, peer support, holistic healing, and alternative treatments, medical) (RAS provider version). |
| 10. | People in recovery can work with health care staff on the development and provision of new programs and services (RAS provider version). |
| 11. | It is important for staff to be knowledgeable about special interest groups and activities in the community (RAS provider version). |

Social responsibility

| 12. | It is not the responsibility of health care providers to support the role of Family and friends in the recovery process of people with mental health problems/illnesses (new). |
| --- | --- |
| 13. | It is not the responsibility of the health care providers to provide opportunities for people with mental health problems/illnesses to have control over their treatment (new) (R). |
| 14. | Health care providers should be entirely responsible about the treatment decisions for people with mental health problems/illnesses (new) (R). |
| 15. | The health care system should involve people with mental health problems/illnesses in the design and implementation of mental health services (new). |
| 16. | Public health programs targeting prevention and early intervention are of little value to people with mental health problems and illnesses (CMHI scale) (R). |
| 17. | Mental health consultation is not a necessary service that should be provided to community caregivers who can help in the care of people with mental health problems and illnesses (CMHI scale) (R). |
| 18. | Social action is required to ensure the success of mental health programs (CMHI scale). |
| 19. | Health care providers should become agents for social change for people with mental health problems and illnesses (CMHI scale). |
| 20. | Collaboration between mental health care providers and non-mental health care providers is relatively unimportant to the success in working with people with mental health problems and illnesses (CMHI scale) (R). |
| 21. | Family, friends and caregivers have their own unique needs that require support from the health care system to help people with mental health problems/illnesses (new). |
| 22. | It is not the responsibility of the health care system to support staff so that they can support and encourage people with mental health problems/illnesses (new) (R). |
| 23. | It is not the role of the health care system to assist in the development of relationships with the community that help people with mental health problems/illnesses (new) (R). |
| 24. | The Emergency room is the best first point of medical care for someone who is having a mental health problem/illness related emergency (new). |
| 25. | The key to managing mental illness is to seek help from medical professionals (OAMI scale). |
| 26. | I would support spending more tax dollars to improve services for the mentally ill (OAMI). |

Social Distance

| 27. | People who have had mental health problems/illnesses are not as trustworthy as the average person (Devaluation/Discrimination scale) (R). |
| --- | --- |
| 28. | Employers should hire people who have mental health problems/illnesses if they are qualified for the job (Devaluation/Discrimination scale). |
| 29. | I feel as comfortable talking to a person with mental health problems/illnesses as I do talking to a person with physical health problems/illnesses (MICA scale). |
| 30. | If my colleague told me they had mental health problems/illness, I would still want to work with them (MICA scale). |
| 31. | People with mental health problems/illnesses are best treated as their diagnosis (new). |
| 32. | People with mental health problems/illnesses should not be given the responsibilities of everyday life (RKI) (R). |
| 33. | I would not go to a physician if I knew they had been treated for a mental illness (R). |
| 34. | I would make close friends with someone who had a mental health problem/ illness (OAMI scale). |
| 35. | I would not want someone with a mental health problems/ illness to be a school teacher (R) (OAMI scale). |

Other dimensions of stigma

| 36 | We need to be protected from people with mental health problems and illnesses (MICA scale) (R). |
| --- | --- |
| 37 | People with mental health problems and illness are responsible for their mental health problems /illnesses (AQ) (R). |
| 38 | People with a mental illness tend to be dangerous and unpredictable (R) (new). |
| 39 | If a person with mental health problems and illnesses complained of physical symptoms such as chest pain, I would think it is because of their mental health problem/illness (MICA scale). |
| 40 | People with mental health problems and illnesses cannot have a good quality of life (AQ) (R). |
| 41 | People with mental health problems/illnesses are lifelong users of the health care system (new) (R). |
| 42 | People with mental health problems/illnesses need help to make their own decisions about their life (new) (R). |
| 43 | Most people with a mental health problems/ illnesses could snap out of it if they wanted to (new) (R). |
| 44 | People with mental health problems/illness are too disabled to work (new) (R). |
| 45 | People with mental health problems/illnesses are often treated unfairly (new). |

Disclosure

| 46 | If I had a mental health problem/ illness, I would never admit this to any of my friends (MICA scale). |
| --- | --- |
| 47 | I would seek help if I thought I had a mental health problem/illness (OAMI scale). |
| 48 | People with mental health problems/illnesses are often treated unfairly (OAMI scale). |
| 49 | If I had a mental health problem/illness, I would never admit this to my colleagues (MICA scale). |
| 50 | If I had mental health problems/illness, I would not be able to live a satisfying life (new) (R). |

(R): reverse scoring required for item.

STARS:

Crowe TP, Deane FP, Oades LG, Caputi P, Morland KG. **Effectiveness of a collaborative recovery training program in Australia in promoting positive views about recovery.** Psychiatr Serv. 2006, **57**(10):1497–500.

RAQ-7:

Borkin JR, Steffen JJ, Ensfield LB et al: **Recovery Attitudes Questionnaire: Development and Evaluation.** Psychiatr Rehabil J 2000, **24**: 95–102.

RAS:

Corrigan PW, Salzer M, Ralph RO, Sangster Y, Keck L: **Examining the factor structure of the recovery assessment scale.** Schizophr Bull 2004, **30**(4):1035–41.

CMHI scale:

Baker F & Schulberg H: **The development of a Community Mental Health Ideology Scale.** Community Mental Health Journal 1967, **3**: 216*–*225.

OAMI scale:

Lillie E, Koller M & Start H: Opening Minds at University: Results of a contact based anti-stigma intervention. Canada; 2010.

Discrimination/Devaluation scale:

Link BG, Cullen FT, Frank J, Wozniak J: **The social rejection of ex-mental patients: understanding why labels matter.** Am J Sociol 1987, **92**:1461–1500.

MICA scale:

Kassam A, Glozier N, Leese M et al: **Development and responsiveness of a scale to measure clinicians' attitudes to people with mental illness (medical student version).** Acta Psychiatr Scand 2010, **122**:153–61

RKI:

Bedregal LE, O'Connell M, Davidson L. **The Recovery Knowledge Inventory: assessment of mental health staff knowledge and attitudes about recovery**. Psychiatr Rehabil J 2006, **30**(2):96–103.

AQ:

Corrigan P, Markowitz FE, Watson A, Rowan D, Kubiak MA: **An attribution model of public discrimination towards persons with mental illness.** J Health Soc Behav 2003, 44:162–179.
