# Supplementary material for: The development and psychometric properties of a new scale to measure mental illness related stigma by health care providers: The opening minds scale for Health Care Providers (OMS-HC)
Source: BMC Psychiatry. 2012 Jun 13;12:62. doi: 10.1186/1471-244X-12-62 (PMC3681304; doi:10.1186/1471-244X-12-62)
Supplement: Additional file 2 — Testing version of the OMS-HC. [file 1471-244X-12-62-S2.docx]

**Additional File 2: Testing version of the OMS-HC**

Item Dimension of stigma Source

| 1. I am more comfortable helping a person who has a physical illness than I am helping a person who has a mental illness. | Social distance | Mental Illness: Clinicians’ Attitudes (MICA) Scale |
| --- | --- | --- |
| 2. If a person with a mental illness complains of physical symptoms (e.g. nausea, back pain or headache), I would likely attribute this to their mental illness. | Diagnostic overshadowing | Mental Illness: Clinicians’ Attitudes (MICA) Scale |
| 3. If a colleague with whom I work told me they had a managed mental illness, I would be as willing to work with him/her. | Social distance | Mental Illness: Clinicians’ Attitudes (MICA) Scale |
| 4. If I were under treatment for a mental illness I would not disclose this to any of my colleagues. | Disclosure | Focus group |
| 5. I would be more inclined to seek help for a mental illness if my treating healthcare provider was not associated with my workplace. | Disclosure | Focus group |
| 6. I would see myself as weak if I had a mental illness and could not fix it myself. | Disclosure | Focus group |
| 7. I would be reluctant to seek help if I had a mental illness. | Disclosure | Focus group |
| 8. Employers should hire a person with a managed mental illness if he/she is the best person for the job. | Recovery | Opinions about Mental Illness Scale |
| 9. I would still go to a physician if I knew that the physician had been treated for a mental illness. | Recovery | Opinions about Mental Illness Scale |
| 10. If I had a mental illness, I would tell my friends. | Disclosure | Mental Illness: Clinicians’ Attitudes (MICA) Scale |
| 11. It is the responsibility of health care providers to inspire hope in people with mental illness. | Social responsibility | Recovery Attitudes Questionnaire |
| 12. Despite my professional beliefs, I have negative reactions towards people who have mental illness. | Social responsibility | Medical Condition regard scale |
| 13. There is little I can do to help people with mental illness. | Social responsibility | Medical Condition regard scale |
| 14. More than half of people with mental illness don’t try hard enough to get better. | Recovery | Opinions about Mental Illness Scale |
| 15. People with mental illness seldom pose a risk to the public. | Dangerousness | Opinions about Mental Illness Scale |
| 16. The best treatment for mental illness is medication. | Social distance | Focus group |
| 17. I would not want a person with a mental illness, even if it were appropriately managed, to work with children. | Social distance | Opinions about Mental Illness Scale |
| 18. Healthcare providers do not need to be advocates for people with mental illness. | Social responsibility | Focus group |
| 19. I would not mind if a person with a mental illness lived next door to me. | Social distance | Opinions about Mental Illness Scale |
| 20. I struggle to feel compassion for a person with a mental illness. | Social responsibility | Medical Condition regard scale |
